# iDetect for vulnerability detection in internet of things operating systems using machine learning

**DOI:** 10.1038/s41598-022-21325-x

**Published:** 2022-10-12

**Authors:** Abdullah Al-Boghdady, Mohammad El-Ramly, Khaled Wassif

**Affiliations:** Department of Computer Sciences, Faculty of Computers and Artificial Intelligence, Cairo University, 5, Ahmed Zewail Street, Dokki, Giza, 12613 Egypt

**Keywords:** Computer science, Software

## Abstract

Internet of Things (IoT) 's devices are ubiquitous and operate in a heterogonous environment with potential security breaches. IoT Operating Systems (IoT OSs) are the backbone software for running such devices. If IoT OSs are vulnerable to security breaches, higher-level security measures may not help. This paper aims to use Machine Learning (ML) to create a tool called iDetect for detecting vulnerabilities in C/C++ source code of IoT OSs. The source code for 16 releases of IoT OSs (RIOT, Contiki, FreeRTOS, Amazon FreeRTOS) and the Software Assurance Reference Dataset (SARD) were used to create a labeled dataset of vulnerable and benign code with the reference being the Common Weakness Enumeration (CWE) vulnerabilities present in IoT OSs. Studies showed that only a subset of CWEs is present in the C/C++ source code of low-end IoT OSs.The labeled dataset was used to train three ML models for vulnerability detection: Random Forest (RF), Convolutional Neural Network (CNN), and Recurrent Neural Network (RNN). The three models were used independently and RF; compared to CNN and RNN, gave the highest accuracy during the testing phase for binary and multiclass classification. RF was chosen as iDetect's ML classifier. Further evaluation was done on an unseen dataset of 322 code snippets taken from TinyOS. iDetect achieved a macro-averaged F1 score (mF1) of 98.5% and weighted-average F1 score (wF1) of 98% for multiclass classification, F1 score (F1) of 97.8% for binary classification, and superior results compared to all three Static Analysis Tools (SATs) used to collect the training dataset.

## Introduction

The IoT is expanding enormously in almost all aspects of modern life with billions of sensors, actuators, and smart devices connected to the Internet. These devices are collecting and exchanging enormous volumes of data about their surroundings. IoT Operating Systems (IoT OSs) are embedded operating systems that run and manage IoT devices, including transferring data over the Internet. Billions of IoT devices are now being connected to the Internet on a daily basis, and their integration into our daily lives has resulted in an Internet of Vulnerabilities^1^. Based on a security report from Forescout Research Labs in 2021^2^, at least 100 million IoT devices are vulnerable to Denial of Service (DoS) and Remote Code Execution (RCE) attacks which allow attackers to take devices offline or take control of them. The number of vulnerabilities reported publically to the Common Vulnerabilities and Exposures database (CVE) increased from 4500 in 2010 to 179,340 in July 2022^3^. According to Gartner's research, IoT systems are the target of more than 25% of cyber-attacks^4^.

Furthermore, many reports addressed large-scale Distributed Denial of Service (DDoS) attacks against IoT devices^5^, while many researches were published to present solutions for IoT vulnerabilities and intrusion detection^6^. In addition, vulnerabilities result from insecurity in the language used and programmers' disregard for secure coding practices^7^. The situation is further complicated by the fact that most IoT OSs are written in C/C++ due to their very powerful low-level programming support. However, at the same time, they are among the least secure programming languages. Some studies claim that 50% of vulnerabilities in open source projects discovered between 2009 and 2019 were in C programs^8^. Hence, securing IoT systems is a critical issue, especially regarding human life, health, or safety.

Our research concentrated on embedded IoT OSs that power low-end IoT devices. Furthermore, most IoT OSs are open source and were developed by people with diverse programming backgrounds and levels of expertise.

The vulnerabilities of IoT OSs are one of the main loopholes that could be exploited for improper use, potentially leading to disasters, most notably in health care applications. As a result, we developed a Machine Learning (ML) model called iDetect, for detecting vulnerabilities in IoT OSs source code written in C/C++ , since it is the dominant language for writing IoT OSs. The main contribution of this research is developing iDetect, and to the best of our knowledge, it is the first tool that uses ML to detect vulnerabilities in IoT OSs. The second contribution of the research is creating a labeled dataset of IoT OS vulnerabilities based on our previous paper's results and findings^9^. Another contribution of this research is comparing three different ML models' ability to detect vulnerabilities.

We experimented with three ML models that had been trained on the final labeled dataset of 5117 code snippets of vulnerable and benign codes covering 54 different types of CWEs^10^. The dataset contains 2626 vulnerable code snippets taken from sixteen releases of four IoT OSs (RIOT, Contiki, FreeRTOS, and Amazon FreeRTOS) and 2491 code snippets of vulnerable and benign codes taken from the Software Assurance Reference Dataset (SARD)^11^.

The rest of this paper is divided as follows: “Background” explains the relevant background knowledge. “Related work” introduces related work on the use of ML for vulnerability detection. “Methodology” presents the research methodology and iDetect design. “Model evaluation and results” presents the research results and evaluation. “Discussion” presents the discussion. Finally, “Conclusion and future work” presents the conclusion and future work.

## Background

The relevant background knowledge about low-end IoT OSs, CWE, and Static Analysis Tools (SATs) was discussed in detail in our previous work^9^. Therefore, this section aims to provide an overview of the remaining relevant background knowledge directly related to this research, mainly machine learning techniques used for vulnerability detection.

### Machine learning

ML is a subset of Artificial Intelligence (AI) that is capable of learning from experiences and historical data to improve the accuracy of outputs without explicit programming. ML is frequently classified based on how an algorithm learns to improve its prediction accuracy. Our study employs three ML algorithms: (1) RF, (2) CNN, and (3) RNN, which are related to both traditional machine learning and Deep Learning (DL). The three algorithms are described briefly in the following subsections.

#### Random forest

RF is a supervised learning algorithm, and it can be used for classification as well as regression. RF is based on the Decision Tree (DT) algorithm that is used in modeling predictions and behavior analysis. It contains many DTs, each representing a unique instance of the RF's classification of data input. The RF algorithm generates multiple decision trees and combines them to produce a more accurate and stable prediction, where the more trees a forest has, the more robust it is. Over-fitting is a problem with deep DT, but it is avoided with RF, which creates trees on random subsets. Because of the large number of DTs involved in the procedure, RF is considered a highly accurate and robust ML model.

#### Convolutional neural network

CNN is a supervised Artificial Neural Network (ANN) that can use an internal data structure such as image data structure and textual data structure with accurate prediction in both image and textual data^12^. CNN requires much less pre-processing than other classification algorithms and can produce better results as the number of training rounds increases. In general, CNNs achieve high accuracy and superior performance when dealing with spatial data^13^.

#### Recurrent neural network

RNN was developed primarily for problems involving time-series or sequential data and sequence prediction^14^. RNN excels in tasks like language translation, speech data prediction, and speech recognition. RNNs are derived from feed-forward neural networks and consist of layers stacked on top of each other, with neurons in each layer. All connections between layers point in the same direction^15^. RNN adds cyclic structure to the network through the self-connection of neurons. Using self-connected neurons, RNN can 'memorize' historical inputs and thus influence network output.

## Related work

This paper proposes a supervised ML-based tool for detecting vulnerabilities in the C/C++ source code of low-end IoT device OSs, focusing on CWEs. To the best of our knowledge, this is the first research that uses ML to detect CWEs vulnerabilities in IoT OSs of low-end devices. Therefore, we have included the influential related works on using ML for vulnerability detection that are close to our research in terms of (1) studying C/C++ and/or (2) using datasets close to ours.

Li et al.^16^ developed a framework called Syntax-based, Semantics-based, and Vector Representations (SySeVR) using a deep-learning classifier based on bidirectional Gated Recurrent Unit (BGRU). They created the dataset using 19 popular C/C++ open source products from National Vulnerability Database (NVD) plus SARD^11^. The framework reached 98% accuracy and 92.6% F1-measure.

Li et al.^17^ developed another deep learning-based vulnerability detector in C source code, called Vulnerability Deep learning-based Locator (VulDeeLocator) which is similar to SySeVR. For the dataset creation, they used NVD and SARD. VulDeeLocator reached 98.8% accuracy and 97.2% F1-measure.

Li et al.^18^ developed a hybrid neural network framework of CNN and RNN for vulnerability detection in C source code. Using the SARD dataset, their hybrid framework achieved 99% accuracy and 98.6% F1-scores. They only used the framework on the SARD, which may have led to bias in the results, and the framework only covered 11 types of CWEs.

Zou et al.^19^ developed a system called multiclass Vulnerability Deep Pecker (μVulDeePecker) based on Deep Learning (DL) which is an extension of their previous work^20^ where they created a system called VulDeePecker. Both works collect the C/C++ vulnerable code dataset from both NVD and SARD. Zou et al. claimed that μVulDeePecker is the first DL-based system for multiclass classification that covers 40 different types of CWEs. μVulDeePecker achieved an mF1 score of 94.22% and a wF1 score of 94.69%. Zou et al. also used μVulDeePecker and VulDeePecker together, achieving an mF1 score of 96.87% and a wF1 score of 96.28%.

However, most of these results are exceptionally high in accuracy, using the same dataset source for training, testing, and evaluation. According to a recent study by Chakraborty et al.^21^, these results should be taken with caution. They replicated the experiments of the most prominent vulnerability detection models, and the results were much less than the originally reported ones. They attributed this to three issues (1) inadequate model, which treats code as a sequence of tokens, ignoring structural and semantic information, (2) irrelevant learning features, and (3) data duplication and data imbalance. We used the lessons learned from this research to avoid some of the pitfalls that negatively impact the quality of vulnerability detection research.

Our research is distinguished from the above, focusing on detecting vulnerabilities in the source code of IoT OSs that run and manage low-end IoT devices with limited resources. We aim to develop and train a model for detecting the most prevalent vulnerabilities in these systems, with CWE taken as our benchmark in the training and prediction phases. Using CWE creates a frame of reference in the developer's mind to clarify the vulnerable code so that s/he can easily handle it. We collected a new dataset of vulnerable C/C++ code snippets focusing on IoT OSs source code from two sources to achieve this goal. The first source is actual real vulnerable code snippets obtained from the source code of sixteen releases of four different IoT OSs up to and including 2020 versions. SARD is the second source, a semi-synthetic and well-documented C/C++ dataset that can be used to create a labeled dataset of benign and vulnerable codes. It was combined with the first source to improve the final labeled dataset and avoid data imbalance, with a total of 5117 code snippets. Furthermore, we used three ML algorithms in training to identify the best model to be exploited in the prediction phase.

## Methodology

**Figure 1:**
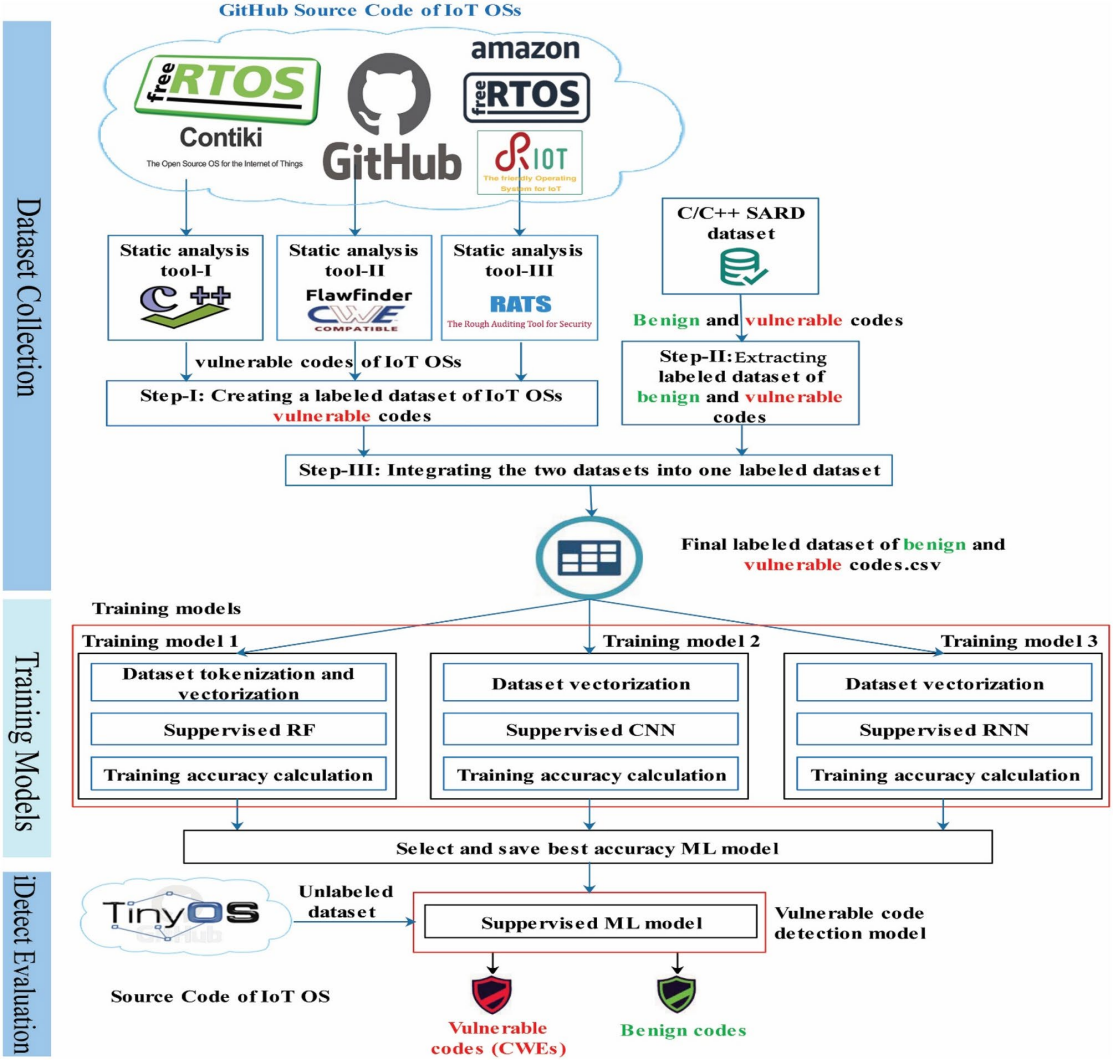
iDetect model development process.

Figure 1 depicts the overall process of developing iDetect model for vulnerability detection in IoT OSs source code. It includes three phases, which are explained in details below. The first phase is building the labeled dataset of benign and vulnerable codes. The second phase is deploying and comparing three training models (Training model 1: supervised RF, Training model 2: supervised CNN, Training model 3: supervised RNN) to select the most accurate one in vulnerable code detection. The third phase is model evaluation on new, never-seen-before data.

### Dataset collection

Our labeled dataset of vulnerable and benign code snippets was created in three steps from two different sources, as shown in Fig. 1. We collected 2626 vulnerable code snippets from IoT OSs using CWEs as a benchmark for identifying and labeling the vulnerabilities, covering 54 types of CWEs discovered in the IoT OSs case study^9^. We added 2491 benign and vulnerable code snippets from SARD relating to the 54 types of CWEs associated with IoT OSs. In total, it includes 5117 code snippets.

For example:

Vulnerable code**: strcpy (message + 7 + strlen (dirent.name), "\"...");**

Description: Does not check for buffer overflows when copying to destination, *strncpy* easily misused, and its Microsoft banned [MS-banned] code,

CWE ID: CWE-120

Vulnerable code presences:

1. Contiki release 2.4\apps\directory\directory.c, line 192.
2. Contiki release 2.7\apps\directory\directory.c, line 192.
3. Contiki release 3.0\apps\directory\directory.c, line 192.
4. Contiki release 3.1\apps\directory\directory.c, line 192.

In step I, and building on our previous work^9^, we used three SATs (Cppcheck version 2.1^22^, Flawfinder version 2.0.11^23^, and Rough Auditing Tool for Security (RATS)^24^) to create a labeled dataset with 2626 snippets of vulnerable codes from sixteen releases of four IoT OSs (RIOT, Contiki, FreeRTOS, and Amazon FreeRTOS) as shown in Table 1. The examples cover all 54 types of CWE found to be common among IoT OS releases^9^. The vulnerable code is labeled according to the type of CWE found.

**Table 1:** The IoT OSs releases used for dataset collection.

IoT OS	Release or Version
RIOT	RIOT R. 2015.09
	RIOT R. 2017.07
	RIOT R. 2019.07
	RIOT R. 2020.04
Contiki	Contiki R. 2.4
	Contiki R. 2.7
	Contiki R. 3.0
	Contiki R. 3.1
FreeRTOS	FreeRTOS v. 6.0.3
	FreeRTOS v. 9.0.0
	FreeRTOS v. 10.0.0
	FreeRTOS v. 10.3.1
Amazon FreeRTOS	Amazon FreeRTOS v. 1.0.0
	Amazon FreeRTOS v. 1.4.0
	Amazon FreeRTOS v. 201908
	Amazon FreeRTOS v. 202007

In step II, we needed to augment the dataset with examples of benign and vulnerable code to avoid data imbalance. For this purpose, we used SARD, a semi-synthetic and well-documented C/C++ database containing both benign and vulnerable code. From SARD, we selected further examples of vulnerable code snippets that have vulnerabilities of the 54 CWE ones found in IoT OSs to further enlarge our dataset and reduce the percentage of false-positive examples (SATs are known to produce some false positives). Additionally, we selected benign code snippets from SARD to balance the dataset. Our SARD's labeled dataset includes 2491 snippets of vulnerable and benign codes.

Step III combines the two labeled datasets into a single labeled dataset and unifies the format. With a total of 5117 vulnerable and benign codes, the final labeled dataset contains 2626 vulnerable code snippets from IoT OSs and 2491 code snippets (538 vulnerable and 1953 benign code snippets) from SARD. The code snippets are the features of the final labeled dataset, where tags are CWEs-ID or Benign code. The data set is made available to researchers to benchmark their work.

### Training models

This phase employs three ML models developed by Python version 3.7.0, TensorFlow version 1.10.0, and Keras libraries on the web-based interactive computing platform of Jupyter Notebook version 5.6.0. We independently applied the multiclass and binary-class classification to the three ML models during this phase. As a result, we made two copies of the final labeled dataset. The first dataset is called Al_Boghdady_Multi_Class, where the code snippets represent the dataset's features, and the CWE types (54 types) and Benign refer to the tags. The second dataset is called Al_Boghdady_Binary, where the code snippets are the dataset's features, and the tags are Vulnerable or Benign code.

We apply multiclass classification for the following reasons: (1) CWE is already used as a benchmark during the vulnerability identification step; (2) Classifying the vulnerable code into CWEs makes it easier for the developer to handle the vulnerable code. We also apply binary classification to compare our work to related works that use binary classification only.

### Model 1: supervised RF

The RF algorithm is based on the DT algorithm, and it generates and combines multiple DT to produce accurate prediction. We trained the RF algorithm using the Scikit-learn (Sklearn) library, which represents the mathematical formulation^25^ of the DT. The DT divides the feature space recursively for a given training vector ${X}_{i}{\in R}^{n}$, $i$=1 to $I$ and a label vector ${\mathrm{y}\in R}^{I}$, so that samples with the same labels or comparable target values are grouped together. Let ${Q}_{m}$ with ${n}_{m}$ samples represent the data at node $m$. Partition the data into ${{Q}_{m}}^{left}\left(\uptheta \right)$ and ${{Q}_{m}}^{right}\left(\uptheta \right)$ subsets for each candidate split $\uptheta =(j, {t}_{m})$ with a feature $j$ and threshold ${t}_{m}$.

$${{Q}_{m}}^{left}\left(\uptheta \right)=\{\left(x,y\right){|x}_{j}\le {t}_{m}\}$$

$${{Q}_{m}}^{right}\left(\uptheta \right)= {Q}_{m}\backslash {{Q}_{m}}^{left}\left(\uptheta \right)$$

The impurity function or loss function $H()$ is used to calculate the quality of a proposed split of node $m$, and then choose the settings that minimizes impurity.

$$G\left({Q}_{m},\uptheta \right)= \frac{{{n}_{m}}^{left}}{{n}_{m}} H \left({{Q}_{m}}^{left}\left(\uptheta \right)\right)+ \frac{{{n}_{m}}^{right}}{{n}_{m}} H \left({{Q}_{m}}^{righ}\left(\uptheta \right)\right)$$

$${\uptheta }^{*}={argmin}_{\uptheta } G\left({Q}_{m},\uptheta \right)$$

Recurs for the subsets ${{Q}_{m}}^{left}\left({\uptheta }^{*}\right)$ and ${{Q}_{m}}^{right}\left({\uptheta }^{*}\right)$ up to the point where ${n}_{m}$< ${min}_{samples}$ or ${n}_{m}$= 1 which indicates the maximum depth permitted. If a target is a classification result taking on values 0, to $K$ -1, with node $m$, let

$${p}_{mk}= \frac{1}{{n}_{m}} {\sum }_{\mathrm{ y}\in {Q}_{m}}I\left(\mathrm{y}=k\right)$$

be the proportion of observations of class $K$ in node $m$. If $m$ is a terminal node, predict proba is set to ${p}_{mk}$ for this region. Because our dataset is not small, the Criterion was applied is 'gini,' which is the function used to evaluate the quality of a split and is represented as follows:

$$H\left({Q}_{m}\right)={\sum }_{\mathrm{ k}}{p}_{mk}\left(1- {p}_{mk}\right)$$

Tokenization is an essential aspect of working with text data, it entails cutting each textual (code snippet in our case) into character substrings known as tokens. Dataset vectorization is the next step. The vector representation we used is called TF-IDF (Term Frequency-Inverse Document Frequency), which is an algorithm based on word statistics for text (code) feature extraction. It associates each document (source code) with an array of size M, with the ith element corresponding to the scaled frequency of the token in the document^26^.

RF parameters are used to either improve the model's predictive ability or make it easier to train, for example, (1) Estimators: number of trees to be built and (2) Criterion: the function for determining a split's quality. The k-fold Cross-Validation (k-fold CV) method was applied to estimate the configuration of a dataset and training performance to determine the mean accuracy of the RF training model. This step was iterated more than 30 times using different training parameters to get the best parameters with the best accuracy. For example, when we applied k-fold to 10, 15, 20, 25, 30, and 35, we discovered that the best accuracy was obtained when we applied k-fold to 20 "the training dataset was divided into 20 non-overlapping folds". We likewise utilized Estimators on 55, 110, 220, and 440 and discovered that the best accuracy was obtained when we used Estimator on 110.

**Figure 2:**
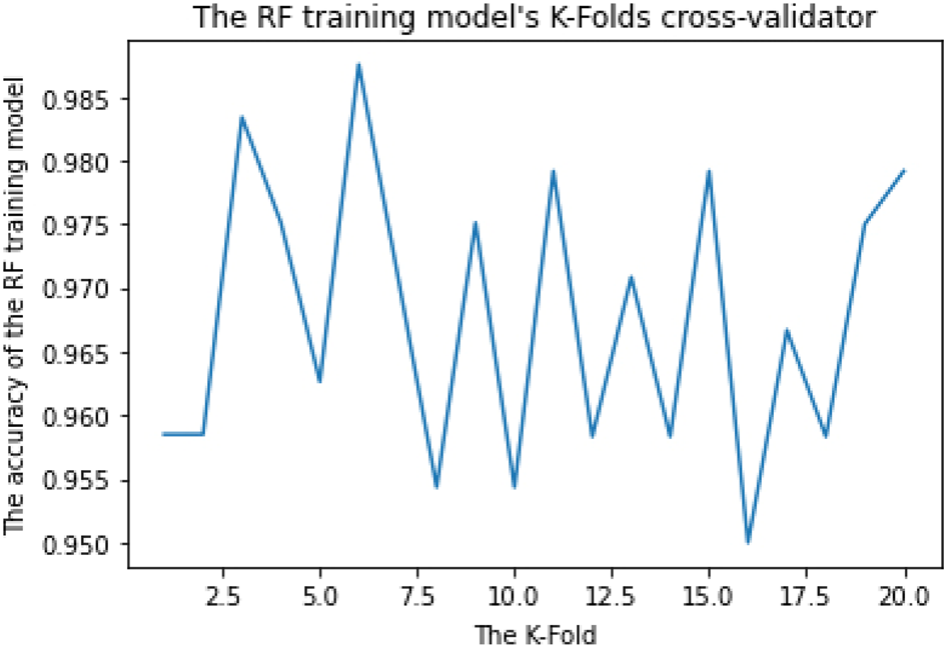
RF training model achieved 96.8% accuracy for multiclass classification.

**Figure 3:**
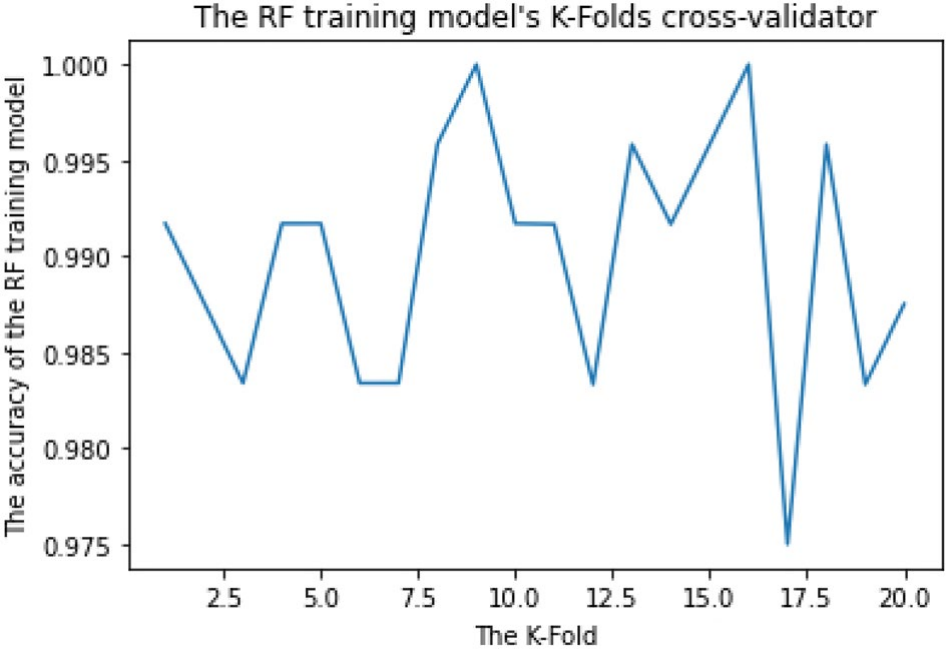
RF training model achieved 99% accuracy for binary-class classification.

As shown in Figs. 2 and 3, the RF training model achieved the mean accuracy of 96.8% and 99% for multiclass and binary-class classification, respectively.

### Model 2: supervised CNN

The first step in the supervised CNN training model is to convert the raw code string into a list of lists "Vectorization". Thirty random states controlling the shuffling were applied to the data before the split (70% for training, 30% for testing). Our CNN training model has 150 input neurons and exploits the layer weight regularization technique (L2)^27^ to improve model generalization. Keras^28^ has an embedding layer for textual data that can be used with neural networks, and it is required for the input data to be integer encoded. Hence, each word is represented by a unique integer.

**Figure 4:**
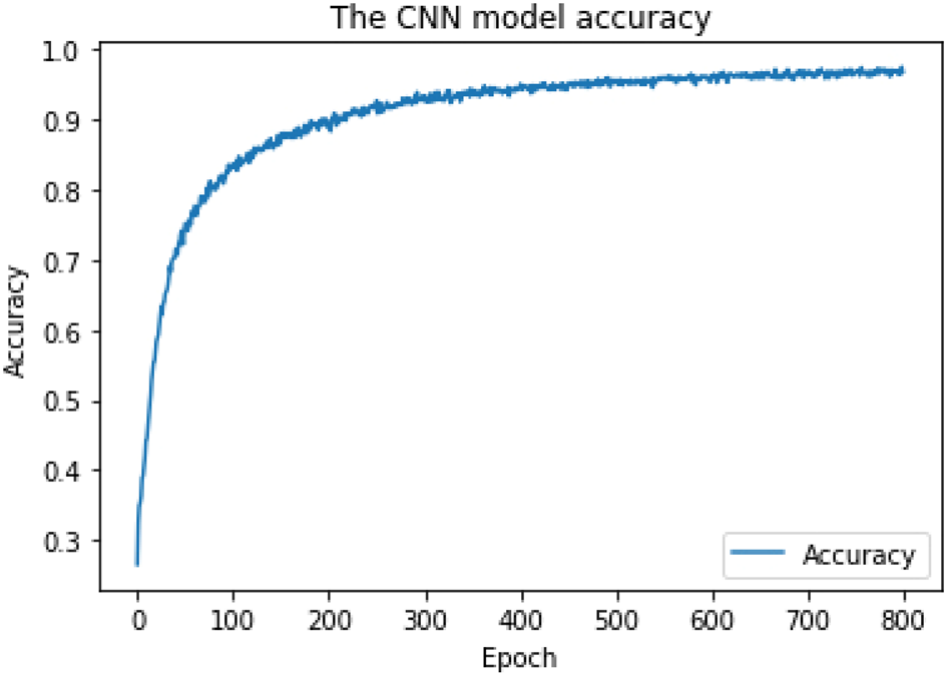
CNN training model achieved 94% accuracy for multiclass classification.

**Figure 5:**
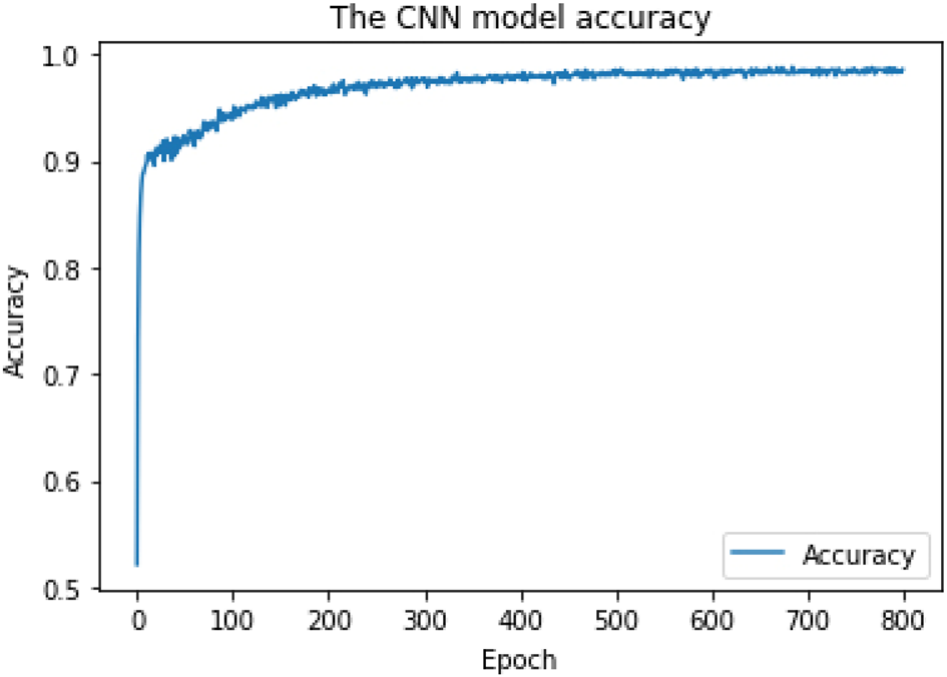
CNN training model achieved 95.8% accuracy for binary-class classification.

The CNN network was constructed as follows: (1) The main input layer with 150 neurons representing the maximum length of a code snippet, (2) One embedding layer with 150 neurons representing each word with a unique integer, (3) Four convolutional layers, (4) Four hidden layers, and (5) The output layer. The Adam optimizer^29^ with the following parameters was applied at the CNN model compilation: (1) learning rate = 1e−4, (2) beta_1 = 0.9, (3) beta_2 = 0.999, and (4) decay = 0. The CNN model was trained over 800 epochs with batch size = 64 batches. For multiclass classification, the output layer applied the "Softmax" activation function, and the output shape = 55 types of (54 types of CWE + Benign). The output layer applied the "Sigmoid" activation function for binary-class classification, and the output shape = 2 types (Vulnerable + Benign). As shown in Figs. 4 and 5, we obtained the final Cross-Validation accuracy of 94% for multiclass classification and 95.8% for binary-class classification, respectively.

### Model 3: supervised RNN

The RNN training model is capable of learning order dependence in sequence prediction problems. The parameters of data split, input layer, embedding layer, hidden layers, output layer, activation functions, epochs, and batches of the supervised RNN training model are the same as those of CNN, but we did not use convolutional layers because they are not part of RNN.

**Figure 6:**
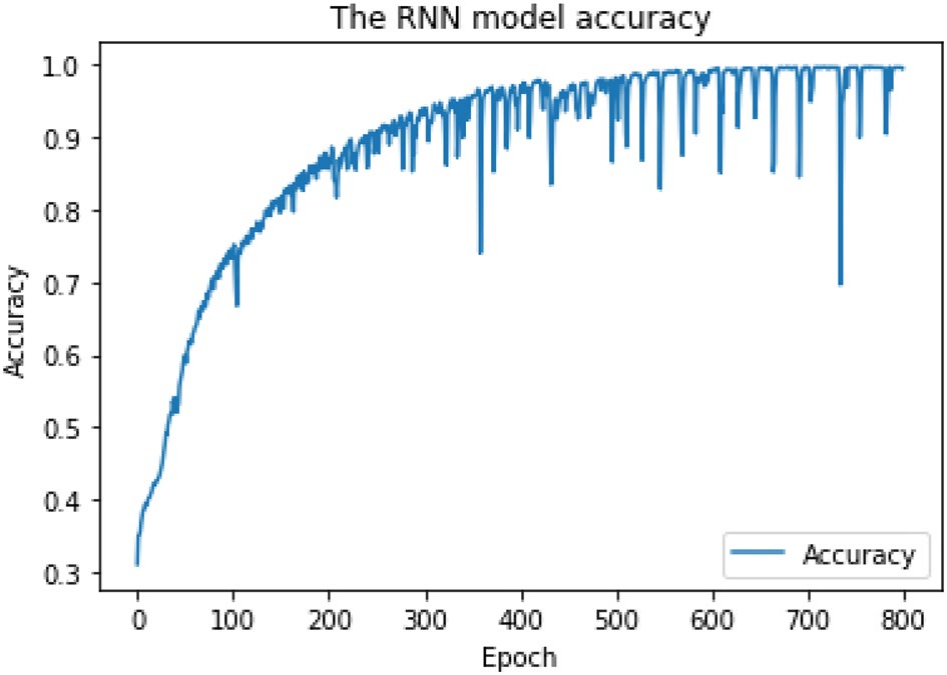
RNN training model achieved 85.6% accuracy for multiclass classification.

**Figure 7:**
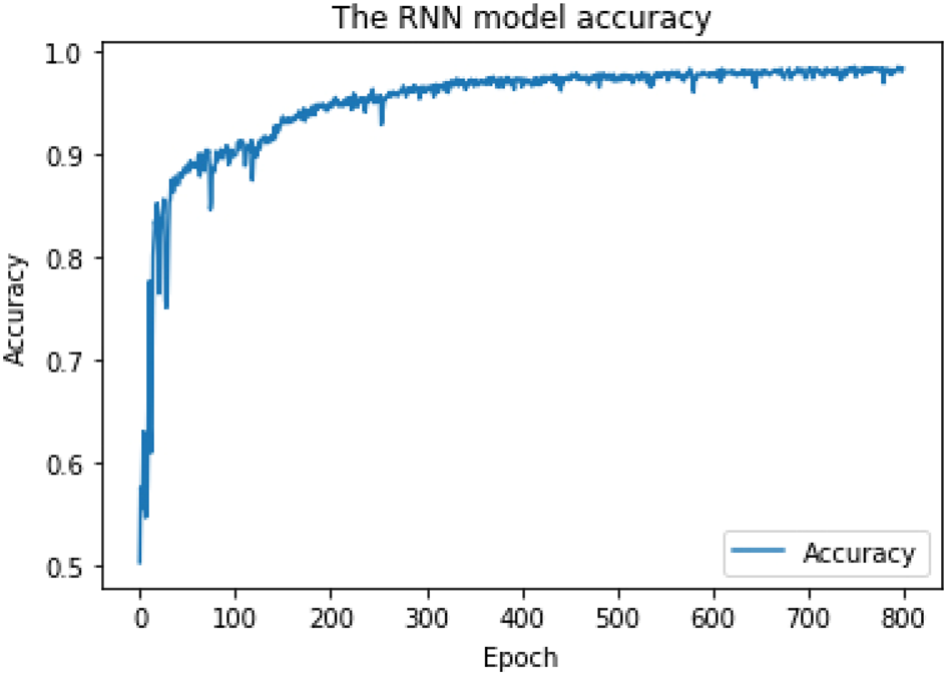
RNN training model achieved 95.7% accuracy for binary-class classification.

As shown in Figs. 6 and 7, the RNN training model achieves final Cross-Validation accuracy of 85.6% for multiclass classification when the "Softmax" activation function is applied to the output layer, and 95.7% for binary-class classification when the "Sigmoid" activation function is applied to the output layer.

The RF model had the highest accuracy of 96.8% for multiclass classification and 99% for binary classification during the training phase, so we chose it as the prediction model for the iDetect tool.

## Model evaluation and results

For evaluation, we chose 322 never-seen code snippets (274 vulnerable codes, 48 benign codes) from other IoT OSs (TinyOS V. 2.1.2) that were not used in data collection, named Tinyos_Evaluation, and they cover 30 different types of code snippets (CWEs or Benign). These snippets were tested and were labeled by the three SATs: Cppcheck, Flawfinder, and RATS. As a result, we can assess iDetect's capability in detecting vulnerable code associated with IoT OSs.

Table 2 summarizes the results and the number of vulnerable codes detected by the three SATs and iDetect for multiclass classification, where Samples is the number of unseen code snippets to be checked. iDetect misses only a small percentage of correct detections. CWE-561, CWE-686, CWE-457, CWE-119, CWE-120, CWE-134, CWE-20, and CWE-126 code snippets were incorrectly reclassified by iDetect. iDetect detects all vulnerable codes and CWE types detected by the three SATs.

**Table 2:** The multiclass classification evaluation results of the three SATs and iDetect on the TinyOS evaluation dataset.

CWE-ID	Samples	Cppcheck	Flawfinder	RATS	iDetect
CWE-561	16	16	0	0	19
CWE-398	37	37	0	0	37
CWE-563	10	10	0	0	10
CWE-686	16	16	0	0	15
CWE-570	7	7	0	0	7
CWE-476	9	9	0	0	9
CWE-571	9	9	0	0	9
CWE-457	10	10	0	0	7
CWE-664	9	9	0	0	9
CWE-783	1	1	0	0	1
CWE-190	5	0	5	0	5
CWE-467	4	4	0	0	4
CWE-682	3	3	0	0	3
CWE-401	1	1	0	0	1
CWE-704	15	15	0	0	15
CWE-119	15	0	15	13	16
CWE-687	7	7	0	0	7
CWE-768	2	2	0	0	2
CWE-120	29	0	29	3	30
CWE-134	14	0	14	5	13
CWE-327	4	0	4	3	4
CWE-78	5	0	2	5	5
CWE-20	15	0	15	9	16
CWE-807	3	0	3	3	3
CWE-350	4	0	0	4	4
CWE-367	4	0	0	4	4
CWE-126	14	0	14	0	13
CWE-362	5	0	5	0	5
CWE-676	1	0	1	0	1
Benign detections	48	166	215	273	48
CWEs detections	274	156	107	49	274

Table 3 summarizes the binary classification results from the three SATs and iDetect. Twelve benign code snippets were incorrectly classified as vulnerable code by iDetect.

**Table 3:** The binary classification evaluation results of the three SATs and iDetect on the TinyOS evaluation dataset.

CWE-ID	Samples	Cppcheck	Flawfinder	RATS	iDetect
Benign code	48	166	215	273	36
Vulnerable code	274	156	107	49	286

### Evaluation metrics

A confusion matrix^30^ is used to evaluate iDetect performance. For binary classification, we used the F1-Score (F1), where the number of vulnerable codes that are correctly detected is referred to as the True Positive (TP). The number of clean codes detected as vulnerable is called the False Positive (FP). True Negative (TN) codes are correctly detected as clean. False Negative (FN) codes are falsely detected as clean. As a result, the False Positive Rate (FPR) = FP/(TN + FP), the False Negative Rate (FNR) = FN/(FN + TP), the Accuracy (Acc) = (TP + TN)/(TP + TN + FP + FN), the Precision (P) = TP/(TP + FP), the Recall (R) = TP/(TP + FN), and the F1 = 2 * [(P*R)/(P + R)].

For multiclass classification, we used the macro F1 score (mF1) and weighted-average F1 score (wF1), where K is the number of different types of code snippets (CWE types and Benign) = 30 types, and N is the number of samples = 322 samples.

$$\mathrm{Average Precision}=\frac{1}{K}\sum_{k=1}^{K}\left({\mathrm{Precision}}_{k}\right)$$

$$\mathrm{Average Recall}=\frac{1}{K}\sum_{k=1}^{K}\left({\mathrm{Recall}}_{k}\right)$$

$$\mathrm{mF}1=\frac{1}{K}\sum_{k=1}^{K}\left( \frac{2*{\mathrm{Precision}}_{k}* {\mathrm{Recall}}_{k}}{{\mathrm{Precision}}_{k}+ {\mathrm{Recall}}_{k}}\right)$$

$$\mathrm{wF}1=\frac{1}{{\sum }_{k=1}^{K}{\mathrm{X}}_{k}}\sum_{k=1}^{K}{\mathrm{X}}_{k}\left( \frac{2*{\mathrm{Precision}}_{k}* {\mathrm{Recall}}_{k}}{{\mathrm{Precision}}_{k}+ {\mathrm{Recall}}_{k}}\right)$$

According to confusion matrix measurement, iDetect archived mF1 = 98.5% and wF1 = 98% for multi-class classification and achieved F1 = 97.8% for binary classification.

## Discussion

The three SATs we used to create the final dataset have many limitations. For example, Cppcheck can detect 83.5% of vulnerabilities and has 7.2% false positives^31^. The results of Flawfinder are close to RATS results, where Flawfinder works by matching simple text patterns, which results in many false positives^28,]^^32^. Thus, we added SARD as the second source to the final dataset because SARD is a semi-synthetic well-documented dataset. We chose it as the second source of our final labeled dataset to (1) enlarge the dataset, (2) reduce the false positives of the three SATs, and (3) balance the dataset.

In the training phase, we independently employed three ML algorithms to select the best accuracy algorithm for the detection model. We iterated the training phase many times with different parameters to obtain the best parameters for the different ML models during the training phase. For example, RF used (90, 100, 110, 120, 130, and 140) for Estimators, which refers to the number of trees to be built. This technique is repeated for the majority of parameters.

The RF algorithm achieved the highest final Cross-Validation accuracy. It achieved 96.8% for multiclass classification and 99% for binary-class classification, requiring the least time for training and prediction. It requires less computational time (about 7 min) for both the training phase and detection phase. Both CNN and RNN require a long time in the training phase to achieve acceptable accuracy and require high computational devices for good performance. As a result, the RF training model was chosen as the classifier for our ML vulnerability detection model, known as iDetect.

Tables 4 and 5 briefly compare our work to related work in multiclass classification and binary classification, respectively.

**Table 4:** The experimental results of multiclass classification between related work tools and iDetect.

Tool name	Dataset	mF1 (%)	wF1 (%)
μVulDeePecker^19^	NVD + SARD	94.22	94.69
μVulDeePecker + VulDeePecker^19^	NVD + SARD	96.87	96.28
iDetect	IoT OSs + SARD	98.5	98

**Table 5:** The experimental results of binary classification between related work tools and iDetect.

Tool name	Dataset	F1 (%)
SySeVR^16^	NVD + SARD	92.6
VulDeeLocator^17^	NVD + SARD	97.2
Hybrid framework^18^	SARD	98.6
iDetect	IoT OSs + SARD	97.8

Except for μVulDeePecker, all related works were for binary classification. μVulDeePecker claim that it is the first DL-based system for multiclass classification, covering 40 types of CWEs. Nonetheless, our work outperforms μVulDeePecker in terms of mF1 and wF1 scores. Our work differs in terms of CWE types and training datasets (except SARD), and our work applies realistic datasets for evaluation. We created a labeled dataset for the training phase from the semi-synthetic dataset "SARD" and the realistic dataset taken from sixteen releases of IoT OSs. For evaluation, we created a realistic dataset from TinyOS V. 2.1.2.

iDetect has some limitations. The IoT OSs used in our work were written using various programming languages such as C, C++ , Python, Perl, Ruby, and Java, although C++ /C is the dominant language. But our dataset (and hence the trained models) only covers examples of C/C++ vulnerable codes. The study depends on non-commercial SATs, which have some limitations regarding the type of vulnerabilities they detect. Therefore, the produced results are limited by the limitations of these tools. While the SATs can find a wide range of CWEs, they are imperfect and may not catch all present vulnerabilities. Hence, we used a combination of SATs to limit this limitation's impact. iDetect is based on static analysis of the source code. Still, there are various insights of vulnerable code detections by utilizing other aspects of the software, such as dynamic analysis of the source code.

## Conclusion and future work

In this work, we built an ML system called iDetect that deploys a trained RF model to detect the vulnerabilities that exist in the C/C++ source code of IoT OSs of low-end devices. We created a labeled dataset from two sources focusing on CWEs most common in IoT OSs. It contains 5117 code snippets taken from sixteen releases of four different IoT OSs for low-end devices from 2010 to 2020 and SARD. SARD was used to balance the data and reduce false positives resulting from SATs. The final dataset contains 54 different types of CWEs plus benign code snippets. We made two copies of the final dataset. The first is the Al_Boghdady_Multi_Class dataset, which was used to train the ML models for multiclass classification. The second copy is the Al_Boghdady_Binary dataset, which was used to train the ML models for binary classification.

The RF training model achieved a multiclass classification accuracy of 96.8% and a binary classification accuracy of 99%. The CNN training model achieved a multiclass classification accuracy of 94% and a binary classification accuracy of 95.8%. The RNN training model achieved a multiclass classification accuracy of 85.6% and a binary classification accuracy of 95.7%. RF achieved the highest accuracy during the training phase and was chosen as our research ML detection model, known as iDetect.

iDetect was evaluated on unseen data taken from TinyOS V. 2.1.2 called Tinyos_Evaluation. iDetect detects vulnerable codes more than any of the other tools alone, and it achieves mF1 = 98.5% and wF1 = 98% for multi-class classification and achieved F1 = 97.8% for binary classification.

The conclusion from this work and our previous one^9^ is that IoT OSs source code contains a specific subset of CWE vulnerabilities, and ML models can provide superior results in vulnerability detection in this limited domain compared to existing SAT tools. Further research is needed that expands this work to cover more IoT OSs, more data, and more languages. But more importantly, these results and tools should be deployed in practical tools to help the unaware developer produce secure and safe IoT systems. Or even better, such tools can be integrated into the DevOps pipeline to raise red flags when vulnerable code is detected before being deployed to IoT systems.

Our immediate future work will extend the final labeled dataset by expanding the use of SATs to identify security errors within IoT OS files written not only by C/C++ but also by other languages such as Python, Perl, and Ruby scripting. In addition, we will exploit more ANN algorithms such as Deep Belief Network (DBN) and Convolutional Deep Belief Network (CDBN) for higher training accuracy and vulnerable code detection. Furthermore, our case study will be extended to include other IoT OSs such as TinyOS, OpenWSN, and Femto OS.

## Data Availability

iDetect and datasets generated during the current study are available from the corresponding author on reasonable request at the following link https://github.com/idetect2022/iDetect.
